# Predictors of Academic and Fieldwork Performance in Occupational Therapy Students: A Systematic Review

**DOI:** 10.1155/2023/7281505

**Published:** 2023-11-25

**Authors:** Yun Wang, Lai-Ha Chung, Chao-Yi Cheng, Wei-Jiun Wang, Li-Chin Chang, Yu-Ming Huang, Sheng-Yuan Tso, Yu-Lin Chen, Ching-Yi Wu

**Affiliations:** ^1^Occupational Therapy Department, Far Eastern Memorial Hospital, New Taipei City, Taiwan; ^2^Department of Occupational Therapy and Healthy Aging Research Center, Chang Gung University, Taoyuan, Taiwan; ^3^Department of Physical Medicine and Rehabilitation, Chang Gung Memorial Hospital at Linkou, Taoyuan, Taiwan

## Abstract

**Introduction:**

Occupational therapy (OT) educational programs are aimed at enrolling a diverse student population that is likely to succeed in the academic and fieldwork components of the program. Comprehending the array of factors that influence students' learning and academic and fieldwork success is important for university educators. This study investigated the existing literature on predictors of academic and fieldwork performance in OT students.

**Methods:**

The search process used in this review included screening, eligibility, and study quality. We searched the PubMed and Cochrane Library databases for literature published in the past 10 years (1 January 2012 to 30 March 2022). As a comprehensive search, the following keywords were used for abstract, title, and keywords sections: occupational therapy student, predictors, fieldwork, academic, academic success, academic performance, fieldwork success, and fieldwork performance. The Medical Education Research Study Quality Instrument was used to assess the quality of studies.

**Results:**

The systematic review retrieved 14 articles that met inclusion criteria. Most were cross-sectional studies, followed by cohort, retrospective analysis of secondary data, and exploratory studies. Four articles focused on academic success, eight focused on fieldwork success, and two explored both aspects. Promising predictors of academic performance included the admission grade point average and the student's approach to studying. Predictors of fieldwork performance included a graduate record examination score, emotional intelligence, and interpersonal relationships.

**Conclusion:**

This systematic review explores predictors of academic and fieldwork success in OT students, which provide opportunities to identify early the learning difficulties of students and assist educators to target modifiable predictors so they can provide high-quality education.

## 1. Introduction

Occupational therapy (OT) educational programs are designed to follow the World Federation of Occupational Therapists (WFOT) Minimum Standards for the Education of Occupational Therapists worldwide. These standards provide a minimum requirement for educational programs and promote educational quality assurance [1]. All OT programs include five components: curriculum content and sequence, educational methods, fieldwork, educators, and educational resources and facilities [1]. Each of these components is developed to be consistent with the philosophy and purpose of the OT profession. Curriculum content is based on contemporary international theories, research findings, and OT practice [1]. Educational methods promote the development of graduate-level knowledge and practice skills, which use multiple information sources, including case studies, experiential learning, and skill training [1]. Fieldwork provides students with practice that promotes the abilities of integrating knowledge, skills, and attitude [1].

OT educational programs are aimed at recruiting a diverse student population that is likely to succeed in the academic (curriculum content) and fieldwork components of the program [1].

Fieldwork education provides students with opportunities to practice new skills, observe patient behavior, and demonstrate professional reasoning so they can gain experience in the occupational therapy role [2]. In addition, students must experience working in diverse settings, including teaching hospitals, institutions, and the community. The duration of the fieldwork practice is a minimum of 1,000 hours [2]. The ability of a student to learn actively in the academic setting and behave positively in fieldwork is crucial for developing knowledge, framework, research capacity, bridging knowledge to clinical reasoning, and maturation of technical skills [3, 4]. Educators who show interest in and provide diverse support for students can break the barriers between supervisors and learners and facilitate the effectiveness of learning [5]. Educators need to understand the factors that influence students' learning processes so they can help guide OT students to successfully accomplish the education programs [6].

Studies on predictors of academic performance in OT are lacking. The main population studied in previous research on academic performance has mostly been nursing and medical students. In nursing courses, previous studies reported that age, sex, and early academic performance were predictors of academic success [7, 8]. Two previous studies of medical courses [9, 10] mentioned that sex, native language, performance at school, learning capacity, and learning style were significant predictors of academic performance. Moreover, other authors have explored the relationship between the learning environment and well-being of the medical students [11]. Similar to nursing and medical courses, studies of undergraduate or master's OT courses reported that age, sex, relationship of living with partners or not, time spent on self-study, how students approached studying, admission grade point average (GPA), and graduate record examination (GRE) scores were prominent predictors of academic performance [3, 12–14]. Bonsaksen et al. [15] also mentioned the learning environment was a possible predictor, although they did not find a significant relationship with learning environment and academic success.

Several studies investigated predictors of fieldwork performance in a variety of healthcare disciplines. McLaughlin et al. [16] and Cheung and Au [17] showed that self-efficacy, personal traits, and anxiety were predictors of fieldwork success in nursing courses. Kupfer et al. [18] and Murden et al. [19] reported that academic performance, personal traits, and empathy were prominent predictors in medical courses. Furthermore, studies of OT courses showed that age, GPA, GRE, level of anxiety, emotional intelligence (EI), personal traits, interpersonal relationships, listening style, resilience, and professionalism were significant predictors of fieldwork success [13, 20–27].

Several systematic reviews investigated the predictors of academic performance to provide advice to educators [11, 28–30]. Pitt et al. [29] categorized the predictors that potentially affected nursing students' academic performance into four sources: demographic, academic, cognitive, and personality/behavioral factors. However, no systematic reviews have been conducted of the factors of fieldwork performance in a nursing course. A study investigating medical students' academic performance classified the predictors into cognitive factors (previous academic ability), noncognitive factors (personality and learning styles), and demographic factors (sex and ethnicity) [30]. Factors to predict fieldwork success in medical course have not been covered in a systematic review. Furthermore, no systematic review about the predictors of OT academic and fieldwork success was found. Few studies that have explored the predictors of OT academic performance identified two sources: sociodemographic factors (such as age, sex, relationships, and time spent on self-study) and cognitive factors (such as admission GPA, GRE, and approaches to studying) [3, 12–15]. In OT fieldwork performance, previous studies mention the predictors from a source of sociodemographic factors (such as age), cognitive factors (such as GPA and GRE), and the personality factors, including anxiety, EI, personal traits, interpersonal relationships, listening styles, and professionalism [13, 20–27].

Establishing the factors that predict academic and fieldwork practice performance is important. Identifying possible predictors will assist educators to effectively plan, design, and implement their curriculum as well as facilitate their ability to underscore the precise strategies needed for different characteristics of students to promote core knowledge and clinical competency [31]. Moreover, this study can provide a review of the predictors of academic and performance success in the OT profession. Determining an overview of predictors can enable the early identification of students at risk of learning difficulties as well as advising educators.

This study investigated the existing literature on predictors of academic and fieldwork success in OT students to provide current evidence-based information to assist educators to target modifiable predictors to provide high-quality education.

## 2. Methods

The search process used in this review was adopted from the suggestions of Siddaway et al. [32]. The process primarily involved searching, screening, eligibility, and study quality.

### 2.1. Data Searches (Searching)

We searched the PubMed and the Cochrane Library databases for articles published in the past 10 years (from 1 January 2012 to 30 March 2022). The hand search also included reference lists and citations of the retrieved articles and the “similar articles” option.

The following key search terms were used: ((“occupational therapy student”) OR (occupational therapy student)) AND ((predictors) OR (academic success) OR (academic performance)), ((“occupational therapy student”) OR (occupational therapy student)) AND ((fieldwork success) OR (fieldwork performance)), and (predictors) AND ((fieldwork) OR (academic)) AND ((“occupational therapy student”) OR (occupational therapy student)).

### 2.2. Selection Criteria (Screening)

The inclusion criteria were (1) full-text articles published in English, (2) study population including OT students, (3) outcomes including the academic performance or fieldwork performance of OT students, and (4) study design including exploratory studies, cohort, secondary data, and cross-sectional studies. Exclusion criteria were studies not relevant to predictors of OT education, including studies relating to nursing and medical training.

### 2.3. Data Extraction (Eligibility)

Using specially developed forms, two reviewers extracted the following information: study design, details of participants, predictive variables, outcomes of academic or fieldwork performance (dependent variables), predictors, Medical Education Research Study Quality Instrument (MERSQI) score, and source of studies (country).

Two independent reviewers initially screened the titles, followed by relevant abstracts and full text, assigned a MERSQI score for each, and calculated a mean quality score across studies. Two reviewers completed the checklist, and disagreements were resolved through discussion to reach consensus.

### 2.4. Quality Assessment (Study Quality)

The MERSQI, which was developed to appraise methodological quality of medical education research, was used to assess the quality of studies. It contains six domains that are based on ten study design and method criteria: study design, number of institutions studied, response rate, data type, internal structure, content validity, criterion validity, appropriateness of data analysis, complexity of analysis, and outcome level. Higher scores indicate higher quality of studies, and the score range was five to 18. In addition, MERSQI has high interrater reliability [33]. Although there were no specific cutoff values to distinguish between high-quality and low-quality study methods, one study established a threshold of 14.0 or higher on the MERSQI score as an a priori cutoff for high quality [34].

## 3. Results

### 3.1. Study Selection

**Figure 1:**
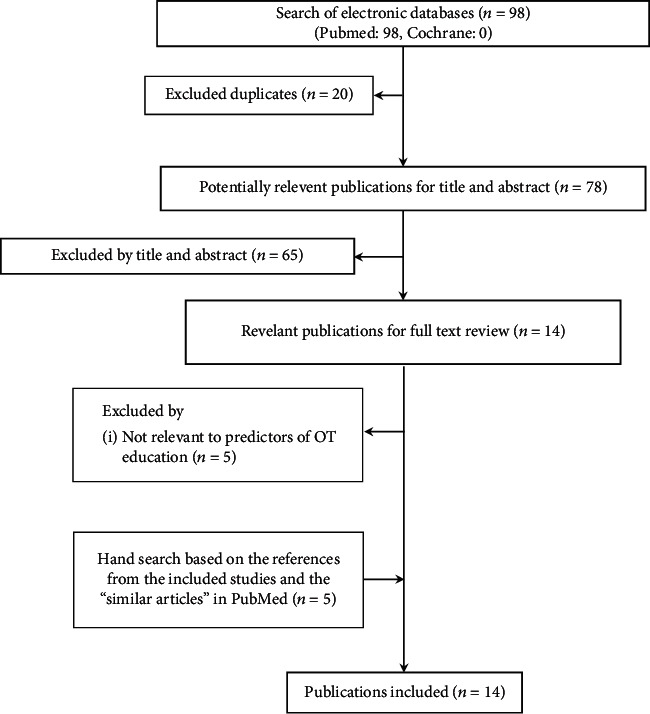
Flowchart of review process.

Our search identified 98 peer-reviewed papers (Figure 1). Of these, we excluded 20 duplicates and 65 not relevant articles by the title and abstract. Finally, 14 articles were included after full-text review and hand search [3, 12–15, 20–27, 35]. Four articles focused on academic success, eight focused on fieldwork success, and two explored both aspects. The main results of the included studies are presented in Table 1.

### 3.2. Characteristics

#### 3.2.1. Study Design

Most of the articles were cross-sectional studies, followed by cohort, retrospective analysis of secondary data, and exploratory studies (Table 1). There were four cross-sectional studies, one retrospective analysis, and one cohort study focusing on academic success in OT. Additionally, there were six cross-sectional studies, one retrospective analysis, one cohort study, and two exploratory studies of fieldwork success in OT.

#### 3.2.2. Participant Details

The 14 studies included in this review represented 2098 participants (range, 49-712). Three studies recruited OT students with master's degrees, and 11 studies recruited undergraduate OT students (Table 1).

#### 3.2.3. Dependent Variables

The outcome measures of academic performance included GPA of the OT program and specific academic courses (physical, cognitive-neurological, psychological-emotional, and communication skills). The outcome measures of fieldwork performance included the Student Placement Evaluation Form (SPEF), American Occupational Therapy Association Fieldwork Performance Evaluation (FWPE), and Team Skill Scale (TSS) (Table 1).

#### 3.2.4. Academic Performance Predictors

Six studies reported the predictors of academic performance in OT students, including age [3, 12], sex [12, 15], living or not with a spouse or partner [3], time spent on self-study activities [12], admission GPA [13, 14], GRE [13], confidential reference [14], and deep and strategic study approaches [12, 15] (Table 1).

Two studies [3, 12] reported that older students had better academic performance than students who were younger. The mature students were more often engaged in other higher education programs before entering the OT program and might thus have better cognitive reserve and the ability to well translate experiences into learning abilities. However, the predictor of age did not show statistical significance in Bonsaksen et al. [15].

The female students had obtained significantly better examination grades compared with the male students [12, 15]. However, sex was not a significant predictor of academic success in Bonsaksen [3]. Results on sex were inconsistent in the previous studies and did not affect like behaviors and environments. Thus, focusing on what can be done to support students' learning is recommended [15].

Living with a spouse or partner was associated with better academic performance compared with single students [3]. Mental health problems and stress were identified as sources of academic failure [3]. Bonsaksen [3] suggested that social support from a partner might serve as a buffer against stress.

The results regarding the time spent on self-study (average hours during a normal week) were inconsistent between two studies [12, 15]. Bonsaksen et al. [12] reported that spending more time on self-study activities was directly associated with higher examination grades. According to their findings, students could improve their academic performance by dedicating more time to study-related materials and tasks. However, Bonsaksen et al. [15] showed no significant correlation between the time spent on self-study and academic performance.

Admission GPA referred to the cumulative average for all undergraduate academic work [14]. Two studies recruiting students with master's degrees in OT [13, 14] found that students with higher admission GPAs achieved higher grades in their OT program. The graduate students may have greater learning abilities and better performance than the undergraduate students because they have already gone through the process of receiving an undergraduate education. Accordingly, they might be able to generalize and apply to similar knowledge of OT subjects. A correlation was also found between self-directed learning and academic achievement, such as the GPA [36]. Self-directed learning may reinforce development of learning autonomy, promote lifelong learning, and prepare competent future health professionals [37].

A significant correlation was found between GRE scores and academic performance, suggesting that higher GRE scores were associated with better academic performance [13]. The GRE measures abilities in verbal reasoning, quantitative reasoning, critical thinking, and analytical writing skills [13]. Students with stronger writing and reasoning abilities might have a greater capacity for learning.

Confidential references were completed by university members familiar with the applicant's academic ability and included with the admission application submitted by students [14]. Higher scores on the confidential references indicated better performance in the OT course's communication skills, potentially identifying students who demonstrated early proficiency in interpersonal relationships [14].

Study approaches, which investigate how the students engaged with study content [12], were measured by Approaches and Study Skill Inventory for Students (ASSIST). The ASSIST assesses three main factors, namely, the deep, strategic, and surface approaches. The deep approach consists of seeking meaning, relating ideas, use of evidence, and interest in ideas. The strategic approach consists of organized study, time management, alertness to assessment demands, achieving, and monitoring effectiveness. The surface approach includes lack of purpose, unrelated memorization, syllabus-focus, and fear of failure [38]. Bonsaksen et al. [12] reported that those who used deep and strategic approaches demonstrated better performance in examination grades, whereas Bonsaksen et al. [15] showed only that the strategic approach had a significant correlation to academic performance. The inconsistent results might be due to the education program designs in different countries. Bonsaksen et al. [12] recruited students from Australia, Singapore, Hong Kong, and Norway, whereas the participants in other Bonsaksen et al. [15] study were only from Norway. OT programs in most countries contained numerous subjects and tests, so the abilities of time management and organized study were important. Curriculum contents usually involved rationales relevant to different fields of OT, evidenced-based, and creativity of intervention designing, which relied on the deep approach strategies. The students who had organized study habits and found meaning in questions generally had great motivation to increase their own understanding. This was directly related to academic and course performance. However, Norwegian OT programs emphasize practice and skill training, which did not fit well with the deep approach [15]. Thus, the strategic study approach might be a promising predictor of academic success in OT programs.

#### 3.2.5. Fieldwork Performance Predictors

The predictors of fieldwork performance in OT were investigated in 10 studies and included age [23], GPA [23], GRE score [13, 25], level of anxiety [23], EI of emotional reasoning and emotional management of others [20, 21], personal traits of extraversion and emotional stability [21], interpersonal relationships of interaction management [24, 27], listening style of sensing [27], resilience [26], and professionalism of equity, enrichment, and altruism [22] (Table 1).

Tan et al. [23] showed that older students had better performance in fieldwork compared with younger students. Specifically, older students demonstrated superior communication skills with clients and significant others [23].

The GPA was calculated as an average of all courses in the OT programs and served as a positive predictor for all areas of OT student fieldwork performance [23]. Students with a higher GPA might have better time management skills, study habits, and overall motivation, which could also contribute to their success in fieldwork [23].

The GRE scores were significantly predictive of OT fieldwork performance [13, 25]. A higher GRE score was associated with better fieldwork performance, suggesting that students with higher general reasoning abilities might be better equipped to demonstrate complex skills during fieldwork training [25].

Anxiety was measured by the Sixteen Personality Factor Questionnaire (16PF). Sources of anxiety for students included feeling inadequate about their knowledge and skills, uncertainties associated with supervisor expectations, and fear of making mistakes and failure [23]. This suggested that students with a certain amount of anxiety might have been more motivated to perform well in fieldwork and, as a result, exerted more effort to achieve their goals [23].

The EI was measured by the Genos Emotional Intelligence Inventory (Genos EI) in two studies [20, 21]. EI means being able to recognize and manage your own emotions and those of others. It also involves using emotions to help with your thinking and being able to understand and control emotions to improve your personal growth [39, 40]. The Genos EI measures how often respondents report EI behavior in the workplace according to seven constructs: emotional self-awareness, emotional expression, emotional awareness of others, emotional reasoning, emotional self-management, emotional management of others, and emotional self-control [40]. Two studies by Brown et al. [20, 21] revealed that higher levels of EI contributed to better performance in OT fieldwork. Emotional reasoning and emotional management were found to be the most indicative factors of fieldwork performance [20, 21]. Communicating with and explaining the intervention plans or home programs to patients showed the importance of emotional reasoning and emotional management. Working in harmony with work colleagues and interdisciplinary teams relied on the abilities of emotional expression, emotional awareness, and emotional management. Understanding of others' emotions had positive effects on building rapport with patients and sustaining professional relationships within interdisciplinary teams [41].

Personal traits, which were defined as enduring characteristics shaping an individual's thoughts, feelings, and interactions with the world [21], were assessed using the Ten-Item Personality Inventory (TIPI) in two studies [20, 21]. The TIPI evaluated five personality constructs: extraversion, agreeableness, conscientiousness, emotional stability, and openness to experience [20]. Traits such as sociability, enthusiasm, and warmth were associated with extraversion and emotional stability, and these characteristics contributed to establishing strong patient relationships and demonstrating empathy in fieldwork performance [21]. While Brown et al. [20] found that personal traits did not predict fieldwork performance, it still strongly suggested further exploration of the relationship between fieldwork performance and personal traits in future studies.

The interpersonal relationships, which encompassed the bonds and interactions that occurred within social connections among two or more individuals, were measured by the Interpersonal Communication Competence Scale (ICCS) [24, 27]. This self-report measure of a person's ability to perform specific skills required to manage interpersonal relationships assesses ten dimensions of interpersonal communication competence: self-disclosure, social relaxation, supportiveness, expressiveness, empathy, assertiveness, environmental control, interaction management, alter centrism, and immediacy [42]. Two studies revealed that the interpersonal relationship dimension of interaction management had positive effects on fieldwork performance [24, 27]. Interaction management referred to one's ability to have effective conversations, such as initiating, terminating, and negotiating, in everyday situations [42]. Great ability of interaction management contributed to a systematic approach to communicate with patients and work colleagues.

Listening, the foundation of all meaningful interpersonal relationships, was linked to the listener's emotional and active involvement in understanding the speaker's thoughts and feelings [27]. The listening styles discussed in two studies [24, 27] were assessed using the Active-Empathic Listening Scale (AELS), a self-report measure of active and empathetic listening that included three different stages of the listening process: sensing, processing, and responding [24]. While Yu et al. [24] found no significant correlations between listening styles and fieldwork performance, Yu et al. [27] demonstrated that higher scores in the sensing listening style were associated with better fieldwork performance. Sensing referred to the capability to perceive, detect, and interpret the underlying conversation. This involved the listener attending to both the implicit and explicit messages and being sensitive to the emotional needs of the speaker [27].

Resilience, which referred to an individual's ability to adapt and withstand challenges, was measured by the Resilience at University (RAU) [26]. RAU is a valid measurement for resilience of undergraduate students, comprising subscales that include finding your calling, living authentically, interacting cooperatively, managing stress, building networks, maintaining perspective, and staying healthy [43]. Managing stress, finding your calling, and living authentically strongly predicted the fieldwork performance [26]. Stress management acted as a protective asset for students, allowing them to use specific strategies such as problem-solving, reflection, and self-care. These strategies contributed to practice education, including increased connectedness, satisfaction, well-being, and employability [26]. Finding your calling and living authentically were the vocational aspects of OT, such as finding a work environment that aligns with one's core values and having a sense of purpose, which were elements of resilience that contributed to students' successful performance outcomes in practice education [26].

Finally, professionalism was measured by the Penn State College of Medicine Professionalism Questionnaire (PSCOPQ), which identifies the elements of accountability, enrichment, equity, honor and integrity, altruism, duty, and respect [44]. The variables of equity, enrichment, and altruism were predictors of OT students' performance in fieldwork [22]. Equity had an impact on the acquisition of crucial skills as students transitioned from university to professional practice. Fairness and equality were essential elements in developing time management skills and the ability to respond appropriately to constructive feedback [22]. Enrichment served as evidence that fieldwork experience contributed to the development of professional gains, including increased assertiveness, supportive communication, improved staff dynamics, and a better understanding of self-care [22]. Altruism was found to foster an altruistic outlook that influenced practice characterized by positive professional relationships, adherence to standards of care, and high levels of career satisfaction [22].

#### 3.2.6. MERSQI Score

Of these 14 articles, the median MERSQI score was 12 (range, 11-13.5) (Table 2): scores were 11 in five studies, 12 in three studies, 12.5 in two studies, 13 in two studies, and 13.5 in two studies. MERSQI scores in all 14 articles were below 14 [34], suggesting that the quality of research exploring predictors of academic and fieldwork performance in OT was still insufficient.

#### 3.2.7. Source of Studies

A total of 14 studies were implemented across seven countries, including Australia (eight studies), Canada (one study), Norway (three studies), Hong Kong (one study), Singapore (one study), the United States (two studies), and England (one study) (Table 1).

### 3.3. Predictors of Academic Success

The authors of six of the included articles reached a consensus on some predictors. Some were only mentioned once in one article, and no consensus in the predictors was reached by all of the authors of these six articles. We attempted to list the predictors that were identified in two or more articles as significantly correlated with academic performance in OT, while excluding the predictors with inconsistent results (Table 3). The categories of predictors of academic success in OT students, which were based on the recommendations of two systematic reviews [29, 30], were grouped into sociodemographic and cognitive factors (Table 3). As a result, the promising predictors of academic success included the cognitive factors of high admission GPA and a strategic study approach.

### 3.4. Predictors of Fieldwork Success

The method of selecting predictors for fieldwork success was the same as selecting predictors for academic success. We listed the predictors reported in ten articles and identified the predictors that had consensus in two or more articles regarding their association with fieldwork success. We also excluded the predictors with inconsistent results (Table 4). The predictors of fieldwork performance in OT were grouped into three factors, including sociodemographic, cognitive, and personality factors, as recommended by two systematic reviews [29, 30]. The promising predictors of fieldwork success included the cognitive factors of GRE score, personality factors of high EI of emotional reasoning and emotional management of others, and interpersonal relationships of interaction management.

## 4. Discussion

This is the first systematic review to examine the predictors of success in academic and fieldwork performance of the profession of OT. This systematic review identified 14 studies and found that the predictors of academic success included high admission GPA and strategic study approach. Additionally, the predictors of fieldwork success included a high GRE score, high EI of emotional reasoning, and interpersonal relationships of interaction management (Tables 3 and 4).

### 4.1. Academic Performance

#### 4.1.1. Educator Issues

Educators consider OT students as successful learners in academic performance because they can fulfill the academic demands of educational programs and possess personal and ethical qualities that enable them to perform effectively as health service providers [14]. However, OT educators themselves bring their own expectations and experiences, which can sometimes impact the students' performance. It is crucial for educators to carefully identify the students who are highly likely to succeed and assess the extent to which they are contributing to the learning issues of students. Two important predictors related to educators are the students' admission GPA and the study approaches advised by the educators.

The process of admitting students into OT programs serves the purpose of identifying individuals who are likely to succeed academically in the programs [14]. Previous studies have shown that considering the students' admission GPA is a reasonable criterion [13, 14]. The results obtained from the previous studies help guide the admission decisions, ensuring that the process is justifiable and beneficial.

Educators should guide students on how to use productive study approaches when learning course content. Additionally, educators should organize curricula, assignments, and examinations in a way that encourages students to actively participate in the learning process using strategic learning approaches, such as organized studying and focusing on achieving success. Educators should discourage students from relying on surface approaches to studying, such as only remembering factual contents, focusing solely on the syllabus, and rote learning [12, 15]. Educators who offer thorough and well-structured teaching materials, along with courses that encourage debating and discussion rather than just memorization, contribute to better academic performance. Bonsaksen et al. [15] also highlighted the benefits of creating student mentoring groups, which provided additional support to students who needed it. These groups allowed successful advanced students to share their perspectives and experiences with less-experienced students, providing valuable guidance and assistance.

#### 4.1.2. Student Issues

To help students succeed in academic performance, it is important to understand the factors that impact their learning process [12]. One important predictor is the approach to studying, which can significantly affect academic achievement [12, 15]. Students should use productive approaches to studying, such as strategic approaches that focus on organized studying and motivation for achievement [15]. Achievement-oriented students are highly dedicated to their studies and motivated to do whatever is necessary to obtain good grades [12]. The advice to students is to focus on striving for achievement by studying to do their best, rather than merely trying to avoid failure.

### 4.2. Fieldwork Performance

#### 4.2.1. Educator Issues

Fieldwork practice is considered influential in shaping students' professional identity as an occupational therapist [20]. For students to succeed in fieldwork, it is important for educators to carefully select students and have a good understanding of the factors that can affect the fieldwork performance of OT students. There are three predictors related to educators: the GRE score, EI of emotional reasoning and emotional management of others, and interpersonal relationships of interaction management.

The GRE score may be a more effective predictor for screening applicants than commonly used indicators such as personal interviews and essays [13]. The GRE is designed to measure the ability to use complex reasoning [25]. The results support considering the GRE scores in admission decision-making, and OT education should be designed to promote clinical reasoning and reflective practice.

The EI abilities of emotional reasoning and emotional management are crucial for OT students since they are expected to engage in effective discussions, collaborate effectively with colleagues and clients, actively participate in workplace communication, and respond positively to constructive feedback [20]. Enhancing the EI skills can be achieved through structured undergraduate training programs that aim to increase students' self-awareness of their own EI and support the development of collaborative skills [20]. Bathje et al. [25] provided some advice, such as using simulated patients to provide immediate feedback, incorporating near-peer learning experiences where senior students offer coaching feedback, and using videotaping of students' performance for self-reflection.

Interaction management plays a significant role in effective communication, an essential skill occupational therapists require to work effectively with clients, their families, and healthcare staff [24]. To enhance students' interaction management, self-management, listening styles, and teamwork communication skills, engaging in evidence-based educational strategies, such as group work, role-play, and problem-based learning is beneficial [27]. Problem-based learning involves group collaboration where students share their opinions and develop a learning plan for themselves and their team. This approach promotes the development of interaction management skills as students learn to manage group conversations, negotiate work assignments effectively, and communicate their ideas respectfully and professionally with their peers [24].

#### 4.2.2. Student Issues

Success in fieldwork for OT students requires mastering technical skills, applying treatment effectively, developing strong clinical reasoning and analytical skills, and establishing a good rapport with clients [25]. EI of emotional reasoning and emotional management of others and interpersonal relationships of interaction management are two key predictors that impact the performance of students in fieldwork.

Having strong skills in emotional reasoning and the ability to manage others' emotions positively impacts communication and fosters a supportive work environment. This understanding of others' feelings promotes a positive atmosphere and motivates individuals to strive for professional achievement [20]. These findings have implications for OT students in fieldwork. OT students are required to possess important skills, such as negotiation, collaboration, and cooperation, and use them appropriately with colleagues and clients. They are also expected to actively engage in workplace communications and demonstrate a positive response to feedback [21], skills that are crucial for professional growth and success of OT students in their fieldwork.

Interaction management refers to the ability to manage one's behavioral procedures in conversation and establish effective communication. It is essential for establishing rapport with clients, identifying potential therapy goals, reporting on a client's status, and implementing therapy plans [24]. For OT students in fieldwork, developing communication competence is crucial, which means being able to adjust their communication approach based on a client's age, sex, diagnosis, cultural background, educational background, and cognitive abilities [27].

This review provides readers with an oversight view of the predictors of academic and fieldwork performance in OT students. Through identification of these predictors, educators might be better equipped to identify the learning difficulties of students and provide effective curriculum designs.

### 4.3. Limitations of the Studies Chosen

This study has several limitations. Firstly, the methodological quality of studies included is insufficient, as indicated by the MERSQI scores, which are below 14 for all 14 articles. Most of the included studies are cross-sectional and exploratory, designs that are easily affected by selection bias. Only one of the 14 studies is a multicenter trial, whereas the remaining studies are single-center trials. Furthermore, the measurement of academic and fieldwork performance lacks standardization across the different studies. For instance, fieldwork performance is measured by SPEF, FWPE, and TSS. SPEF and FWPE contain more dimensions than TSS, leading to challenges for comparisons among studies. Lastly, non-English articles were not included.

## 5. Conclusion

This systematic review explores multiple predictors of academic and fieldwork performance in OT students. In academic performance, admission GPA and study approaches are promising predictors. Moreover, GRE scores, EI, and interpersonal relationships are emerging predictors of fieldwork performance. These predictors provide opportunities to identify early the learning difficulties of students, as well as assist educators to target modifiable predictors to provide high-quality education. However, the overall quality of these studies is insufficient, highlighting the need for more high-quality OT education research.

## Figures and Tables

**Table 1:** Methods of included studies.

Author	Study design	Population (sample size)	Predictive variables	Dependent variables	Predictors	MERSQI score	Country
Academic performance							
Bonsaksen [3]	Cross-sectional study	Undergraduate OT students (*N* = 123)	(1) Sociodemographic factors: age, sex, parents' education level, parents' type of education (2) Relationship factors: living with spouse or partner (3) Education factors: education priority, previous higher education experience, average weekly hours of self-studies 4. Work factors: average weekly hours of paid work	Academic performance	Academic performance: age and participants who lived with a spouse/partner	11	Norway
Bonsaksen et al. [12]	Cross-sectional study	Undergraduate OT students (*N* = 712)	(1) Sociodemographic factors: age, sex, prior higher education, and time spent on self-study (2) ASSIST: deep, strategic, and surface approaches	GPA	GPA: age, sex, time spent on self-study, and deep and strategic approaches	13	Australia, Hong Kong, Norway, Singapore
Lysaght et al. [14]	Retrospective analysis	New master in OT students (*N* = 129)	(1) Admission GPA (2) Confidential reference (3) Background experience: physical sciences, social sciences, and experience working with vulnerable populations	(1) GPA (2) Specific academic courses: physical, cognitive-neurological, psychological-emotional, and communication skills	GPA: admission GPA Communication skills: confidential reference	11	Canada
Bonsaksen et al. [15]	Cross-sectional study	OT first-year students (*N* = 174)	(1) Sociodemographic factors: age, sex, educational priority, prior higher education, and time spent on self-study (2) CEQ (3) ASSIST: deep, strategic, and surface approaches	GPA	GPA: sex and strategic approach	13.5	Norway

Fieldwork performance							
Brown et al. [20]	Cross-sectional study	OT second- and third-year students (*N* = 114)	(1) Genos EI: ESA, EEX, EAO, ER, ESM, EMO, and ESC (2) TIPI	SPEF	SPEF: EAO, ER, EMO, and EEX	13	Australia
Brown et al. [21]	Cross-sectional study	OT first- and second-year students (*N* = 114)	(1) Genos EI: ESA, EEX, EAO, ER, ESM, EMO, and ESC (2) TIPI: extraversion, agreeableness, conscientiousness, emotional stability, and openness to experience	TSS	TSS: ER, EMO, extraversion, and emotional stability	11	Australia
Brown et al. [22]	Exploratory study	OT third- and fourth- year students (*N* = 135)	PSCOPQ: accountability, enrichment, equity, honor and integrity, altruism, duty, and respect	SPEF	SPEF: equity, enrichment, and altruism	13.5	Australia
Tan et al. [23]	Exploratory study	OT third-year students (*N* = 49)	(1) Age (2) Sex (3) GPA (4) 16PF	SPEF	SPEF: age, GPA, and 16PF	12	Australia
Yu et al. [24]	Cross-sectional study	OT third- and fourth-year students (*N* = 70)	(1) ICCS: self-disclosure, social relaxation, supportiveness, expressiveness, empathy, assertiveness, environmental control, interaction management, alter centrism, and immediacy (2) LSP-R (3) AELS	SPEF	SPEF: interaction management	12	Australia
Bathje et al. [25]	Retrospective analysis of secondary data	Master of Science degree in OT (*N* = 115)	(1) GRE (2) GPA	FWPE	FWPE: GRE	12	United States
Brown et al. [26]	Cross-sectional study	OT third- and fourth- year students (*N* = 135)	(1) RAU: finding your calling, living authentically, interacting cooperatively, managing stress, building networks, maintaining perspective, staying healthy (2) RSA	SPEF-R	SPEF-R: managing stress, finding your calling, and living authentically	12.5	Australia
Yu et al. [27]	Cross-sectional study	OT second-, third-, and fourth-year students (*N* = 132)	(1) AELS (2) LSP-R (3) ICCS: self-disclosure, social relaxation, supportiveness, expressiveness, empathy, assertiveness, environmental control, interaction management, alter centrism, and immediacy	SPEF-R	SPEF-R: interaction management and AELS	12.5	Australia

Academic and fieldwork performance							
Kirchner et al. [13]	Cross-sectional study	Master's degrees in OT student (*N* = 63)	(1) Admitted GPA (2) GRE (3) Essay	(1) GPA (2) Level II fieldwork score	GPA: admission GPA and GRE	11	United States
Howard and Jerosch-Herold [35]	Three consecutive cohorts	OT or physiotherapy students BSc degree (*N* = 168)	Entry score	(1) Academic score (2) Fieldwork score	Entry qualifications are poor predictors	11	England

*Note.* OT: occupational therapy; GPA: grade point average; ASSIST: Approaches and Study Skill Inventory for Students; CEQ: Course Experience Questionnaire; 16PF: Sixteen Personality Factor Questionnaire; SPEF: Student Placement Evaluation Form; SPEF-R: Student Placement Evaluation Form-revised; GRE: graduate record examination scores; FWPE: AOTA Fieldwork Performance Evaluation; Genos EI: Genos Emotional Intelligence Inventory; ESA: emotional self-awareness; EEX: emotional expression; EAO: emotional awareness of others; ER: emotional reasoning; ESM: emotional self-management; EMO: emotional management of others; ESC: emotional self-control; TIPI: Ten-Item Personality Inventory; TSS: Team Skill Scale; ICCS: Interpersonal Communication Competence Scale; LSP-R: Listening Style Profile; AELS: Active-Empathic Listening Scale; RAU: Resilience at University; RSA: Resilience Scale for Adults; PSCOPQ: Penn State College of Medicine Professionalism Questionnaire.

**Table 2:** MERSQI scale.

First author, year	MERSQI scale						
	Study design	Sampling	Type of data	Validity of evaluation instrument	Data analysis	Outcomes	Total score
Bonsaksen, 2016 [3]	1	1	3	1	3	2	11
Bonsaksen, 2017 [12]	1	2	3	2	3	2	13
Lysaght, 2009 [14]	1	1	3	1	3	2	11
Bonsaksen, 2021 [15]	1	2.5	3	2	3	2	13.5
Brown, 2016 [20]	1	2	3	2	3	2	13
Brown, 2017 [21]	1	2	1	2	3	2	11
Brown, 2020 [22]	1	2.5	3	2	3	2	13.5
Tan, 2004 [23]	1	1	3	2	3	2	12
Yu, 2018 [24]	1	1	3	2	3	2	12
Bathje, 2014 [25]	1	1	3	2	3	2	12
Brown, 2020 [26]	1	1.5	3	2	3	2	12.5
Yu, 2019 [27]	1	1.5	3	2	3	2	12.5
Kirchner, 2001 [13]	1	1	3	1	3	2	11
Howard, 2000 [35]	1	1	3	1	3	2	11
Median score (range) of 14 articles	12 (11 to 13.5)						

*Note.* MERSQI: Medical Education Research Study Quality Instrument.

**Table 3:** Summary of review findings: predictors of academic performance in occupational therapy students.

Predictors of academic performance	Outcome	Frequency of studies reporting association	Studies	Mean MERSQI score
Sociodemographic factors				
Age	(i) Greater outcomes with older students	2/3	[3], [12]	12
	(ii) No significance	1/3	[15]	13.5
Sex	(i) Greater outcomes with women	2/3	[12], [15]	13.25
	(ii) No significance	1/3	[3]	11
Living with spouse or partner or not	Greater outcomes with living with spouse or partner	1/1	[3]	11
Time spent on self-study	(i) Greater outcomes with more time engaged in self-study activities	1/2	[12]	13
	(ii) No significance	1/2	[15]	13.5
Cognitive factors				
Admitted GPA	Greater outcomes with higher admission GPA	2/2	[13], [14]	11
GRE	Greater outcomes with higher GRE	1/1	[13]	11
Confidential reference	Greater outcomes with higher scores of confidential reference	1/1	[14]	11
Study approach	(i) Greater outcomes with deep and strategic approaches	1/2	[12]	13
	(ii) Greater outcomes with strategic approach	1/2	[15]	13.5

*Note.* GPA: grade point average; GRE: graduate record examination scores; MERSQI: Medical Education Research Study Quality Instrument; CEQ: Course Experience Questionnaire.

**Table 4:** Summary of review findings: predictors of fieldwork performance in occupational therapy students.

Predictors of fieldwork performance	Outcome	Frequency of studies reporting association	Studies	Mean MERSQI score
Sociodemographic factors				
Age	Greater outcomes with older students	1/1	[23]	12
Cognitive factors				
GPA	(i) Greater outcomes with higher GPA	1/2	[23]	12
	(ii) No significance	1/2	[25]	12
GRE	Greater outcomes with higher GRE	2/2	[13], [25]	11.5
Personality factors				
16PF (anxiety)	Greater outcomes with high level of anxiety	1/1	[23]	12
Genos EI	Greater outcomes with high level of EI	2/2	[20], [21]	12
TIPI (personal traits)	(i) Greater outcomes with high level of extraversion and emotional stability	1/2	[21]	11
	(ii) No significance	1/2	[20]	13
ICCS (interpersonal relationships)	Greater outcomes with high level of interaction management	2/2	[24], [27]	12.25
AELS (listening style)	(i) Greater outcomes with higher scores of sensing	1/2	[27]	12.5
	(ii) No significance	1/2	[24]	12
Resilience	Greater outcomes with higher resilience	1/1	[26]	12.5
PSCOPQ (professionalism)	Greater outcomes with high level of equity, enrichment, and altruism	1/1	[22]	12.5

*Note.* GPA: grade point average; 16PF: Sixteen Personality Factor Questionnaire; Genos EI: Genos Emotional Intelligence Inventory; TIPI: Ten-Item Personality Inventory; ICCS: Interpersonal Communication Competence Scale; AELS: Active-Empathic Listening Scale; PSCOPQ: Penn State College of Medicine Professionalism Questionnaire; MERSQI: Medical Education Research Study Quality Instrument.

## Data Availability

The data of this systematic review used to support the findings of this study were included within the article.
